# Combined clinical significance of MRI and serum mannose-binding lectin in the prediction of spinal tuberculosis

**DOI:** 10.1186/s12879-024-09462-2

**Published:** 2024-06-22

**Authors:** Fei Qi, Lei Luo, Chuangye Qu, Weibing Bao, Wenqi Wang, Xiaozhong Zhu, Dengjiang Wu

**Affiliations:** 1 Department of Orthopedics, Wuhan Hankou Hospital, Wuhan, 430000 Hubei Province China; 2 Department of Orthopedics, Guangzhou Huaxin Orthopaedic Hospital, Shantou University, Guangzhou, 510630 Guangdong Province China; 3 Department of Orthopedics, Lanzhou Petrochemical General Hospital (The Fourth Affiliated Hospital of Gansu University of Traditional Chinese Medicine), Lanzhou, 730060 Gansu Province China; 4 Department of Radiology, Gansu Provincial Hospital of Traditional Chinese Medicine (The First Affiliated Hospital of Gansu University of Traditional Chinese Medicine), No. 418 Guazhou Road, Qilihe District, Lanzhou, 730050 Gansu Province China; 5 Department of Orthopedics, The Fifth Affiliated Hospital of Guangzhou Medical University, No. 621, Gangwan Road, Huangpu District, Guangzhou City, 510700 Guangdong Province China

**Keywords:** Spinal tuberculosis, MBL, MRI, Combined diagnosis

## Abstract

**Background:**

Spinal tuberculosis (STB) is a local manifestation of systemic infection caused by Mycobacterium tuberculosis, accounting for a significant proportion of joint tuberculosis cases. This study aimed to explore the diagnostic value of MRI combined with mannose-binding lectin (MBL) for STB.

**Methods:**

124 patients suspected of having STB were collected and divided into STB and non-STB groups according to their pathological diagnosis. Serum MBL levels were measured using ELISA and a Pearson analysis was constructed to determine the correlation between MBL and STB. ROC was plotted to analyze their diagnostic value for STB. All the subjects included in the study underwent an MRI.

**Results:**

The sensitivity of MRI for the diagnosis of STB was 84.38% and specificity was 86.67%. The serum MBL levels of the patients in the STB group were significantly lower than the levels in the non-STB group. ROC analysis results indicated that serum MBL’s area under the curve (AUC) for diagnosis of STB was 0.836, with a sensitivity of 82.3% and a specificity was 77.4%. The sensitivity of MRI combined with MBL diagnosis was 96.61%, and the specificity was 92.31%, indicating that combining the two diagnostic methods was more effective than using either one alone.

**Conclusions:**

Both MRI and MBL had certain diagnostic values for STB, but their combined use resulted in a diagnostic accuracy than either one alone.

## Background

Tuberculosis, a widespread global infectious disease, is caused by Mycobacterium tuberculosis [1]. Spinal tuberculosis (STB) accounts for about 2% of all tuberculosis cases, about 15% of extra-pulmonary tuberculosis, and 50% of all bone tuberculosis, making it the most common form of bone and joint tuberculosis [2]. Among all STB cases, cervical tuberculosis accounts for 4.4%, thoracic for 40.6%, and lumbar for 51.7% [2]. STB is prevalent in the adolescent population and tends to shift towards middle-aged and elderly age groups [3]. Its onset and the atypical symptoms at the early stage can easy to be confused with septic spondylitis, osteoporotic vertebral compression fracture, various kinds of spinal primary tumors or metastatic tumors, eosinophilic granuloma and other diseases. This confusion can lead to diagnostic errors, increasing the pain of the patients and delaying the treatment [4, 5]. Moreover, if the disease worsens, it may result in nerve function damage, and spinal deformity, leading to muscle weakness, sensory loss, spinal scoliosis, scoliosis, and even limb paralysis [6, 7]. Therefore, to diagnose STB more accurately, it is especially important to understand the diagnostic value of each diagnostic technique.

The gold standard for the diagnosis of STB is detecting Mycobacterium tuberculosis in patient samples through smears and/or cultures [8]. However, the lengthy process and low positivity rate of bacterial culture make it unsuitable for early diagnosis and treatment. In addition, distinguishing Mycobacterium tuberculosis from other bacterial granulomatous lesions on pathologic examination can be challenging, increasing the difficulty of diagnosis [4]. Currently, STB is mainly diagnosed by a combination of clinical manifestations, laboratory tests, imaging tests, and pathologic biopsies [9]. Imaging examination includes X-ray, computed tomography, magnetic resonance imaging (MRI), and ultrasound [10]. MRI is the most commonly used in the clinic, which can clearly show various manifestations of STB, such as bone destruction, intervertebral space narrowing, paravertebral abscess, and vertebral canal involvement, to detect STB in early stage and treat it in early stage [11].

In recent years, hematological examination has been applied more and more in the early diagnosis of spinal tuberculosis, among which the immunological diagnosis of tuberculosis bacillus has been highly praised, which has advantages of rapid, economic and other advantages, and the diagnostic efficiency is worthy of recognition. Mannose-binding lectin (MBL) is an innate immune protein produced by the liver and secreted into the bloodstream. In contrast to its proposed deleterious role in other infections such as invasive pneumococcal disease [12], invasive aspergillosis [13] or bronchiectasis [14], MBL deficiency may be advantageous in preventing tuberculosis by limiting uptake into macrophages. Serum MBL level can be influenced by its gene mutation, and plays a regulatory role in tuberculosis immunity [15]. Therefore, its role in the development of STB was examined in the current study.

This study aims to investigate the diagnostic value of MBL and MRI for diagnosing STB. In addition, the diagnostic accuracy of the combination of the two methods was further explored, so as to improve the understanding and diagnostic level of STB among clinical workers.

## Methods

### Recruitment of patients

Patients with STB diagnosed clinically or pathologically in General Hospital of Lanzhou Petrochemical Company from September 2018 to May 2023 were selected. The patients who were screened by the inclusion and exclusion criteria were included in the study. All volunteers provided the informed consent. The protocols of this article were approved by the ethics committee of General Hospital of Lanzhou Petrochemical Company and adhere to the tenets of the Declaration of Helsinki.

All patients were confirmed or ruled out by means of Mycobacterium tuberculosis culture or pathological biopsy. Inclusion criteria included: (1) complete case data; (2) MRI imaging data; (3) all examinations were within 5 days before surgery; (4) agreed to participate in the study. Patients with incomplete case data or not cooperating with relevant examinations were excluded.

Blood was withdrawn from all included individuals after fasting for more than 8 h. Serum samples were obtained after natural coagulation and centrifugation at room temperature. All samples were prepared for MBL detection.

### Detection of all patients by MRI

The imaging data obtained in this part of the experiment were analyzed and determined by two senior imaging physicians. If the results of the two physicians did not agree, a third radiologist determined the results. The instrument used for the MRI examination was an Aera 1.5T (Siemens, Erlangen, Germany). The patient underwent a plain scan and an enhancement scan in sequence. The examination was performed with the help of a body orthogonal coil, and the patient was scanned in sagittal, coronal, and transverse positions. The T1-weighted parameters were set to TR 400–600 ms, TE 15–30 ms. T2-weighted imaging parameters were 2500–3500 ms and 95 ms for TR and TE, respectively. The DWI sequence was set as 2400 ms for TR, 68 ms for TE, and a b-value of 0, 800 s/mm^2^. The scanning matrix was set to 256 × 256, the layer thicknesses were all 3 mm, the layer spacing was 1 mm, and the time was set to 10–16 s. Before the enhancement scans, the patient’s collecting vein was injected with gadopentetate dimeglumine (0.2 mmol/kg) (Gd-DTPA; Bayer Healthcare, Berlin, Germany) at a rate of 2 ml/s using a high-pressure syringe (Nemoto, Tokyo, Japan). Enhancement scans were performed using sequential T1WI fat-suppressed sequences for 120–180 s.

### Determination of MBL

Ten microliters of the sample to be tested were added to the microplate and incubated at 37 degrees for 30 min. The enzyme-labeled reagent was added to the microplate and the same warm bath was performed for 30 min. The chromogen was added, the termination solution was added after 15 min in the dark, and absorbance values were determined at a wavelength of 450 nm.

### Observation indicators

All individuals were confirmed or excluded from STB by pathological examination. The significance of MRI, MBL levels, and the combined diagnosis of the two was assessed using pathological findings as criteria.

### Statistical analysis

Statistical analysis was performed using SPSS 21.0 and GraphPad 7.0. The differences between the non-STB and STB groups were determined using the independent student T test or χ2 test. The correlations between MBL and clinical information were unveiled by Pearson correlation. The receiver operating characteristic curve (ROC) was drawn to research the diagnostic significance of MBL levels. *P* < 0.05 was considered statistically significant.

## Results

### Basic information data of all volunteers

The average age and gender distribution of the non-STB and STB groups are presented in Table 1, and no significant difference was observed (*P* > 0.05). Levels of C-reactive protein (CRP), lipopolysaccharide-binding protein (LBP), white blood cells (WBC), erythrocyte sedimentation rate (ESR), and lymphocyte were elevated in patients with STB compared to those with non-STB (*P* < 0.05, Table 1). However, no difference was identified in neutrophils between the non-STB group and the STB group (*P* > 0.05, Table 1).

**Table 1:** The discrepancy of clinical characteristics between non-STB group and STB group

Indicator	Non-STB *N* = 62	STB *N* = 62	*P*
Age, year	44.14 ± 9.59	44.09 ± 10.37	0.978
Gender			0.470
Male, n	37	32	
Female, n	25	30	
CRP, mg/L	1.22 ± 0.59	23.05 ± 11.11	< 0.001
LBP, ng/mL	84.03 ± 12.96	94.15 ± 20.49	0.001
WBC, ×10^9^/L	5.86 ± 1.16	6.89 ± 1.33	0.020
ESR, mm/h	9.11 ± 3.9	36.33 ± 15.08	< 0.001
Neutrophil, %	61.32 ± 4.33	63.78 ± 9.16	0.059
Lymphocyte, %	24.16 ± 6.82	29.03 ± 10.12	0.002

Annotation: STB, spinal tuberculosis; CRP, C-reactive protein; LBP, lipopolysaccharide-binding protein; WBC, white blood cells; ESR, erythrocyte sedimentation rate

### Diagnostic value of MRI

Out of the STB-positive patients, 54 (87.10%) had positive MRI test results, and 8 (12.9%) had incorrect tests. In the non-STB group, the number of those with correct MRI detection was 52 (83.87%); the number of those with incorrect MRI detection was 10 (16.13%). The sensitivity of the MRI test was 84.38% and the specificity was 86.67%.

### Correlations of MBL and clinical characteristics

**Fig. 1:**
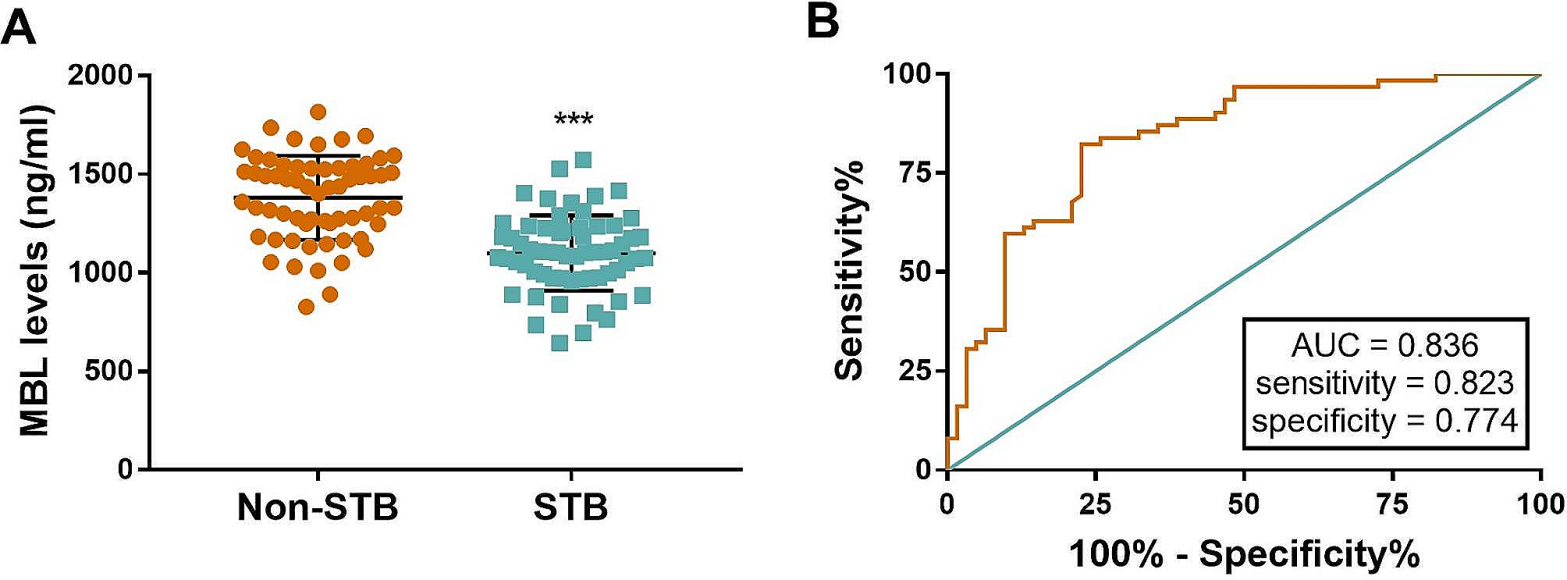
Concentration and predictive possibility of MBL. **(A)** Decreased levels of MBL in the STB group. **(B)** Diagnostic significance of MBL. ***P < 0.001, compared to the non-STB group

Serum MBL level in STB patients was 1100 ± 24.33 ng/ml and that of the non-STB group was 1380 ± 27.04 ng/ml. The difference in MBL level between the two groups was significant (*P* < 0.001, Fig. 1A).

We examined the correlation between MBL and clinical inflammation-related indicators in STB, a condition triggered by a viral infection. Our findings showed inverse correlations between MBL and CRP, LBP, WBC, ESR, neutrophil, and lymphocyte (*P* < 0.001, Table 2).

**Table 2:** Correlations between indictors and MBL

Indicator	Correlation coefficient (*R*)	*P*
CRP, mg/L	-0.794	< 0.001
LBP, ng/mL	-0.659	< 0.001
WBC, ×10^9^/L	-0.488	< 0.001
ESR, mm/h	-0.571	< 0.001
Neutrophil, %	-0.558	< 0.001
Lymphocyte, %	-0.592	< 0.001

Annotation: MBL, mannose-binding lectin; CRP, C-reactive protein; LBP, lipopolysaccharide-binding protein; WBC, white blood cells; ESR, erythrocyte sedimentation rate

### Diagnostic significance of MBL

After plotting the ROC graph, the AUC area was 0.836. indicating that MBL has diagnostic value (Fig. 1B). The optimal Yoden index of 0.597, with the sensitivity of 0.823 and the specificity of 0.774, at which point the MBL cut-off value was 1243 ng/ml. Of the 62 STB patients, 51 were correctly identified as positive by MBL concentration, while 11 were incorrectly identified as negative, yielding an accuracy rate of 82.26%. Among 62 non-STB patients, the number of MBL diagnosed incorrectly was 14 were incorrectly diagnosed by MBL, whereas 48 were correctly diagnosed, giving an accuracy rate of 77.42%.

### Combined diagnosis of MRI and MBL

The combined diagnosis of MRI and MBL levels were further certificated. Their combination represented a certain predictive potential with a sensitivity of 96.61% and a specificity of 92.31%. The rate of joint diagnosis was 91.94% in STB patients, numbering 57. In non-STB patients, the correct rate of joint diagnosis was 96.77% and the number was 60. The positive predictive value (PPV), negative predictive value (NPV), positive likelihood ratio (PLR), and negative likelihood ratio (NLR) of MBL, MRI, and joint diagnosis are exhibited in Table 3. The PLR and NLR of combination diagnosis were 28.50 and 0.08, respectively, indicating that the likelihood of diagnosing or ruling out STB was high (Table 3).

**Table 3:** The diagnostic value of MBL, MRI, and their combination

Indicator	PPV	NPV	Sensitivity	Specificity	PLR	NLR
MBL	78.46%	81.36%	82.26%	77.42%	3.64	0.23
MRI	84.38%	86.67%	87.10%	83.87%	5.40	0.15
Combination	96.61%	92.31%	91.94%	96.77%	28.50	0.08

Annotation: PPV, positive predictive value; NPV, negative predictive value; PLR, positive likelihood ratio; NLR, negative likelihood ratio

## Discussion

Tuberculosis is a globally prevalent infectious disease. While pulmonary tuberculosis is the most common but several parts of the body can be infected by Mycobacterium tuberculosis, such as the spine [16, 17]. Early diagnosis of STB poses a challenge for clinicians due to its atypical early clinical manifestations and the low sensitivity of laboratory tests [18]. Therefore, STB is often missed or misdiagnosed, resulting in the inevitable emergence of multiple complications and even spinal deformities in the later stages of disease development. Early diagnosis, accurate diagnosis, and related differential diagnosis of STB patients are particularly urgent.

X-ray is the fastest imaging method for the diagnosis of STB, which can intuitively understand the early pathological changes of the spine caused by tuberculosis [19]. However, in the early stage of STB, X-rays generally show no abnormal signs [20]. Therefore, the diagnosis of STB should be supplemented by other imaging tests, such as CT and MRI, on the basis of X-ray [21]. MRI can image tissues with multiple sequences, has good soft tissue resolution, and is more sensitive to changes in water and protein components, especially for tumors invading spinal cord, nerve roots, dural and other structures, which can help better clinical identification [22]. CT can accurately identify bone lesions, especially osteolytic lesions, and then observe abnormalities in the vertebral space and body based on reconstruction technology [23]. In short, the biggest advantage of CT examination is that it can clearly show the lesion, and the ability to distinguish bone is better than MRI. For atypical STB, further MRI examination is required before surgery to determine the lesions in the spinal canal.

MRI is often used for the diagnosing of STB due to its sensitivity in the early detection [24, 25]. It outperforms other imaging techniques, clearly displaying early vertebral inflammation and slight swelling of paravertebral soft tissues [26]. It can also determine the extent and nature of intra-vertebral canal lesion invasion, especially Gd-DTPAZ enhancement scan [27]. Tuberculosis in the vertebral body presents as a low signal on T1WI and a high signal on T2WI, which helps differentiate degenerative changes and spinal infections, thus reducing chances of misdiagnosis [28]. However, the diagnostic accuracy of MRI can be limited due to symptoms similarities between STB and other spinal disorders [29]. Therefore, it is necessary to combine MRI with other diagnostic methods when diagnosing STB. In this study, based on the pathological diagnosis results, the MRI results of the included population were analyzed, and it was found that the sensitivity, specificity, positive predictive value, and negative predictive value of the diagnosis of STB were all good, but there were still missed diagnosis and misdiagnosis rates, which reflected that MRI examination could not fully meet the requirements of early diagnosis of STB.

MBL is an innate immune protein that activates the immune response through macrophage-specific recognition and participates in the innate immune response, which is the first barrier against infections [30]. MBL can specifically recognize and bind complex glycan structures on pathogens and have potential as antiviral and antibacterial agents. After tuberculosis infection, the body stress promotes the production of MBL, and then participates in inflammation and immune response. The lack of MBL in patients with ankylosing spondylitis increases the chance of tuberculosis infection, suggesting a correlation between MBL and tuberculosis [31]. Results of a meta-analysis show that serum MBL levels are significantly lower in patients with pulmonary tuberculosis than in healthy controls and may be a potential diagnostic marker [32]. MBL is also a substantial complement component, and its depletion inhibits the remodeling process of bone healing [33]. Infection of the spine by tuberculosis bacilli will inevitably cause damage to the spinal corpus and paravertebral structures [34].

In the present study, serum MBL levels were reduced in patients with confirmed STB, suggesting that the development of STB may be accompanied by the reduction of serum MBL. The expression of MBL is limited in STB patients, which hinders the normal immune response of the body. Therefore, the low expression of MBL can be considered to be closely related to the pathogenesis of STB and can be used for the early diagnosis of STB. Reversely, the occurrence of STB may have affected MBL levels through both immune effects and bone destruction. MBL was inversely associated with CRP, LBP, WBC, ESR, neutrophils, and lymphocytes, reflecting that MBL was linked with the suppressed inflammatory responses of STB. In addition, the ROC results found that the level of MBL has some clinical value in the diagnosis of STB patients. Due to the STB diagnostic errors present in MRI, the measurement of MBL was chosen in this study to reduce the misdiagnosis and missed diagnosis associated with MRI diagnosis. The results showed that the combination of MBL and MRI can raise the diagnostic sensitivity and specificity to over 90%, which is higher than the single test, suggesting that MBL can be used as a complement to MRI for the diagnosis of STB. However, the results of this study have the drawbacks of a small sample size, single research center, and lack of other joint indexes. In the future, other studies with larger sample sizes are needed for the results verification. In addition, the dysregulation of MBL can be detected in various diseases, such as sepsis, cardiovascular diseases, pneumonia and so on [35]. Therefore, in clinical diagnosis, it is necessary to make a comprehensive judgment combined with clinical symptoms.

## Conclusions

In conclusion, MBL was negatively correlated with the inflammation of STB. Both single MBL and MRI offer some diagnostic value in STB patients. When used together, they enhance the diagnostic accuracy of MRI, providing highly beneficial in diagnosing STB. This article is necessary to provide a prompt, reliable, and comprehensive diagnostic foundation for clinicians, helping to prevent treatment delays and reduce the occurrence of spinal deformity.

## Data Availability

The datasets used and/or analysed during the current study are available from the corresponding author on reasonable request.
